# Web-Based Mindfulness Meditation as an Adjunct to Internet-Delivered Cognitive Behavioral Therapy for Public Safety Personnel: Mixed Methods Feasibility Evaluation Study

**DOI:** 10.2196/54132

**Published:** 2024-01-30

**Authors:** Caeleigh A Landry, Hugh C McCall, Janine D Beahm, Nickolai Titov, Blake Dear, R Nicholas Carleton, Heather D Hadjistavropoulos

**Affiliations:** 1 Department of Psychology University of Regina Regina, SK Canada; 2 PSPNET University of Regina Regina, SK Canada; 3 Canadian Institute for Public Safety Research and Treatment Regina, SK Canada; 4 eCentreClinic School of Psychological Sciences Macquarie University Sydney Australia

**Keywords:** public safety personnel, PSP, internet therapy, mindfulness, meditation, internet-delivered cognitive behavioral therapy, iCBT

## Abstract

**Background:**

Public safety personnel (PSP) are individuals who work to ensure the safety and security of communities (eg, correctional workers, firefighters, paramedics, and police officers). PSP have a high risk of developing mental disorders and face unique barriers to traditional mental health treatments. The PSP Wellbeing Course is a transdiagnostic, internet-delivered cognitive behavioral therapy (iCBT) course tailored to assist PSP with symptoms of depression, anxiety, and posttraumatic stress disorder (PTSD). The initial course outcomes are promising, but some clients report some challenges with learning skills and recommend adding additional resources. Mindfulness meditations, which help people to experience the world and their reactions to the world in open and nonjudgmental ways, may complement the existing PSP Wellbeing Course.

**Objective:**

This study aims to examine the feasibility of mindfulness meditations in iCBT tailored for PSP. Information was gathered to evaluate engagement and client experiences with mindfulness meditations, symptom change, and the relationship between mindfulness meditation use and symptom change.

**Methods:**

A mixed methods study was conducted on PSP enrolled in the PSP Wellbeing Course who were offered 5 mindfulness meditations during the program (ie, 1/lesson). Clients completed questionnaires on depression, anxiety, PTSD, anger, insomnia, resilience, and mindfulness at pretreatment and at 8 weeks; an 8-week treatment satisfaction questionnaire; and brief weekly measures of mindfulness meditation engagement. We used paired sample *t* tests (2-tailed) to assess changes in outcomes over time and partial correlations to assess whether mindfulness meditation use predicted outcomes at posttreatment. A total of 12 clients were interviewed about their perceptions of the mindfulness meditations, and interviews were analyzed using directed content analysis.

**Results:**

Among the 40 clients enrolled, 27 (68%) reported using the mindfulness meditations, practicing for an average of 4.8 (SD 8.1) minutes each week. Most interviewees described the mindfulness meditations as beneficial but also reported challenges, such as discomfort while sitting with their feelings. Clients provided suggestions for better integration of mindfulness into iCBT. Overall, clients who completed the PSP Wellbeing Course with mindfulness meditations experienced statistically significant improvements in symptoms of anxiety (*P*=.001), depression (*P*=.001), PTSD (*P*=.001), and anger (*P*=.001) but not insomnia (*P*=.02). Clients also experienced improvements in resilience (*P*=.01) and mindfulness (*P*=.001). Self-reported time spent meditating was not associated with changes in symptoms over time.

**Conclusions:**

This study provides new insight into the integration of mindfulness meditations with iCBT for PSP. It demonstrates the partial feasibility of adding mindfulness meditations to iCBT, revealing that some, but not all, PSP engaged with the meditations and reported benefits. PSP reported using the mindfulness meditations inconsistently and described challenges with the meditations. Improvements can be made to better integrate mindfulness meditation into iCBT, including offering mindfulness meditation as an optional resource, providing more psychoeducation on managing challenges, and offering shorter meditations.

## Introduction

### Background

Public safety personnel (PSP; eg, border services officers, correctional workers, firefighters, Indigenous emergency managers, operational intelligence personnel, paramedics, police officers, public safety communicators, and search and rescue personnel) have greater exposure to potentially psychologically traumatic events than the general population and a greater risk for several psychological disorders. This heightened risk is, in part, attributable to the nature of their vocations [1-3]. In a recent pan-Canadian study, 44.5% of PSP screened positive for at least 1 mental disorder on a set of questionnaire measures [1], which is far higher than the rate of mental disorder diagnoses in the general population (ie, 10% [4]). Other studies show PSP also report high levels of anger [5] and sleep difficulties [6]. Despite the significant need for mental health treatment, PSP face several unique barriers to treatment as a function of their vocational cultures and requirements [7]. PSP report concerns about engaging in face-to-face mental health treatment options, including geographical barriers; the cost of treatment; difficulty navigating services; long wait times; and, in particular, concerns about privacy, confidentiality, and stigma [6,8-10].

Internet-delivered cognitive behavioral therapy (iCBT) appears to be well positioned to address barriers to treatment experienced by PSP, as it delivers treatment materials in a private and accessible web-based format. There is strong evidence that iCBT has comparable effectiveness with face-to-face cognitive behavioral therapy (CBT) among the general population [11]. A recent observational trial found that iCBT tailored specifically for PSP was associated with large pre-post reductions in symptoms of generalized anxiety disorder, major depressive disorder, and posttraumatic stress disorder (PTSD), although it was not compared with a control condition [9]. In written feedback, PSP who participated in iCBT reported appreciating several aspects of iCBT, including the format and content, the accessibility of the course, the additional resources and examples, and the therapist guidance [12]. Nevertheless, some PSP also reported challenges with learning skills [13] and provided suggestions for improving iCBT, including adding more resources [12].

The addition of mindfulness resources represents a promising addition to iCBT with PSP, given the growing research attention on the benefits of mindfulness [14-16] and its use in CBT [17]. Kabat-Zinn [18] provided the most commonly used definition of mindfulness: a three-component definition that calls for paying attention (1) on purpose, (2) in the present, and (3) nonjudgmentally. Mindfulness shifts awareness to focus on present-moment activities, thoughts, and sensations, which over time improves emotion regulation and, in turn, reduces symptoms [19]. Mindfulness may be cultivated through numerous activities that bring individuals into the present moment and into their experience without judgment. Mindfulness meditation, in particular, is a practice to cultivate mindfulness and refers to the practice of silently observing one’s own internal and external environment without attempting to change anything [19]. In the general population, several studies have shown that web-based mindfulness programs result in small but significant improvements in symptoms such as depression and anxiety [14-16]. In terms of PSP, mindfulness-based interventions have been associated with reductions in stress, insomnia, burnout, anger, anxiety, and depression [20,21]; however, research has been limited to pilot or feasibility studies, police populations, and in-person class delivery style [3,22].

Recent literature suggested that incorporating mindfulness may enhance the benefits derived from traditional therapies [17]. Mindfulness practice has been successfully integrated within face-to-face interventions, such as mindfulness-based stress reduction, mindfulness-based cognitive therapy, and acceptance and commitment therapy [17]. Moreover, a recent systematic review and meta-analysis reported that mindfulness-enhanced iCBT has demonstrated significant reductions in anxiety and depression in clinical populations, above and beyond comparison conditions [17], but to date, no research has focused on offering web-based mindfulness-enhanced iCBT for PSP. Although it is possible that the results of mindfulness-enhanced iCBT will generalize to the use of iCBT for PSP, this remains an empirical question. Some past research among PSP suggests that they may be more skeptical of mental health support [7]. Some studies on mindfulness among PSP have yielded mixed results, with only some participants reporting improvements [21]. Therefore, it is important to explore whether mindfulness meditations will be used and positively evaluated by PSP within the context of iCBT and whether adding mindfulness to iCBT will positively or negatively impact engagement, satisfaction, and outcomes. Adding mindfulness meditation to iCBT has the potential to increase the engagement, satisfaction, and effectiveness of iCBT. However, adding additional components could also reduce effectiveness through additional burden placed on users or if users have negative views of mindfulness meditations.

### Objectives

As a first step in understanding the use of mindfulness meditation as part of iCBT for PSP, this study was designed to examine the feasibility of adding mindfulness meditations to an existing iCBT program for PSP, called the PSP Wellbeing Course, by (1) evaluating the level of engagement with the mindfulness meditations (eg, practice length and frequency) as well as with the intervention; (2) assessing client experiences with mindfulness meditations during and after treatment; (3) measuring changes in measures of anxiety, depression, and PTSD to compare outcomes with previously published outcomes of the PSP Wellbeing Course [9]; and (4) assessing the relationship between mindfulness meditation use and outcome measures. We hypothesized that clients would actively participate in the mindfulness meditations, report positive experiences, and also identify areas for improvement in the mindfulness meditations. We also hypothesized that clients would report statistically significant improvements in symptoms of anxiety, depression, and PTSD and that greater mindfulness meditation use (ie, length and frequency) would predict less severe self-reported symptoms at posttreatment [23]. This feasibility study is the first step in assessing whether mindfulness meditations will be deemed usable and acceptable in iCBT for PSP.

## Methods

### Ethical Considerations

This study was approved by the Research Ethics Board of the University of Regina (2019-157). Clients were made aware of the details of the study and the potential risks and benefits of participating, and they provided informed consent before participation. Clients were given access to the PSP Wellbeing Course but were not otherwise offered incentives to encourage participation. Client data were stored on a secure server and deidentified before analyses.

### Clients and Procedure

PSP were informed of the PSP Wellbeing Course offered by PSPNET through presentations, emails distributed through PSP organizations, social media, and word of mouth and encouraged to visit the PSPNET website [24]. PSPNET is a clinical research unit based at the University of Regina that develops, delivers, and conducts research on iCBT for PSP. In addition to offering a therapist-guided PSP Wellbeing Course, PSPNET also offers a therapist-guided PSP PTSD Course and a self-guided version of the PSP Wellbeing Course, all of which were available for PSP to select from at the time they visited the website. Prospective clients who were interested in the PSP Wellbeing Course read about the program and completed a consent form and a brief web-based screening questionnaire. Once they completed the web-based screening, they scheduled and completed a phone screening with a trained clinician. To be eligible for the intervention and thereby this study, clients needed to be current or past PSP (career or volunteer), be residing in an eligible Canadian province or territory (at the time of this study, this included Alberta, New Brunswick, Nova Scotia, Ontario, Prince Edward Island, Saskatchewan, and Nunavut), be aged at least 18 years, have access to an internet connection, and be willing to provide an emergency medical contact. Prospective clients were ineligible and referred to other services as appropriate if they reported a high suicide risk; reported a past-year suicide attempt or suicidality-related hospitalization; reported a primary problem with psychosis, mania, or substance use; or reported current involvement in another psychological treatment. The eligibility criteria were assessed during the web-based screening and the subsequent phone screening. Suicide risk was first assessed using item 9 from the Patient Health Questionnaire-9 (PHQ-9) [25], which inquires about suicidal ideation. Clinicians then conducted a clinical interview by phone to assess suicide risk, including asking about past-year attempts and hospitalizations. Severe alcohol or drug problems were assessed using validated questionnaires (ie, scored ≥20 on the Alcohol Use Disorder Identification Test [26] or ≥25 on the Drug Use Disorder Identification Test [27]). Psychosis and mania were assessed based on clinical history but were not reported by any client. Eligible clients were enrolled in the intervention and assigned to therapists who were either registered master’s-level social workers or registered psychologists. Therapists would email or call clients once or twice a week, depending on client preference. Therapist support was designed to help clients work on skills within the course, apply the skills to their lives, and troubleshoot difficulties. Clients were asked to complete regular symptom measures throughout the course on a weekly basis and at 8 weeks. At 10 weeks, the first 30 clients were invited to complete an interview assessing their perspectives on the mindfulness meditations and the course in general. There were 12 clients who agreed to participate in the interviews.

### Materials

#### The iCBT Intervention

The PSP Wellbeing Course is a transdiagnostic iCBT course for PSP adapted from a previous Canadian iCBT course, the Wellbeing Course, which was initially developed at the Macquarie University in Australia and has been successful in treating a range of symptoms in Australia [28-31] and Canada [32-35]. More information on the origins of the intervention [25] and the adaptations of the intervention [29-32] can be found elsewhere. The course uses a theoretical, pragmatic approach to treatment that teaches clients skills that can be applied to various symptom presentations. The intervention specifically includes five core lessons: (1) introduction of CBT and identifying symptoms, (2) monitoring and challenging automatic thoughts, (3) management of physical symptoms, (4) graded exposure, and (5) goal setting and relapse prevention. Lessons are presented in a slideshow format and include text, diagrams, and case stories about PSP. Clients can download materials, homework assignments, and supplementary information on many topics (eg, panic, assertiveness, sleep, and grief). The intervention is designed to be completed in 8 weeks, but clients can have access to a therapist for up to 16 weeks and to the course materials for up to 1 year. Clients receive automated emails encouraging them to work through the materials during this period.

#### Mindfulness Meditations

Unlike the previous versions of the PSP Wellbeing Course, the version used in this study included a downloadable, guided, audio mindfulness meditation for each of the 5 lessons of the intervention and psychoeducational material on mindfulness meditations before the first meditation. Each lesson included a different type of mindfulness meditation designed to complement the CBT skills taught in that lesson and was based on evidence of successful implementation in other programs [22,36-39]:

- Lesson 1: grounding (eg, turning attention to physical sensations)
- Lesson 2: loving kindness (eg, cultivating feelings of love for self and others)
- Lesson 3: awareness of breath (eg, focusing on breathing slowly and deeply)
- Lesson 4: awareness of the 5 senses (eg, cultivating awareness of the environment through the 5 senses)
- Lesson 5: body scan (eg, focusing on the body for areas of tension).

Each mindfulness meditation was audio-recorded by a voice actor and was designed to be approximately 10 minutes long, consistent with previous recommendations [39-41]. Each mindfulness meditation was accompanied by a text script that clients could read in lieu of the audio. All clients who enrolled in the PSP Wellbeing Course were provided with access to the mindfulness meditations. Clients were encouraged to complete 10 minutes of mindfulness meditation practice per day while completing the intervention.

### Measures

#### Overview

During eligibility screening, we administered a questionnaire to assess demographic and occupational characteristics. We also asked about pretreatment engagement in a mindfulness meditation practice. At both pretreatment and 8 weeks postenrollment, consistent with past research on the PSP Wellbeing Course*,* we administered the following measures: the PHQ-9 to measure symptoms of depression [25], the Generalized Anxiety Disorder-7 (GAD-7) to measure symptoms of generalized anxiety [42], and the Posttraumatic Stress Disorder Checklist for the *DSM-5* (PCL-5) to measure symptoms of PTSD [43]. As anger and sleep problems are common among PSP [5,6], we also administered the Dimensions of Anger Reaction Scale-5 (DAR-5) to measure problems related to anger [44] and the Insomnia Severity Index (ISI) to measure sleep difficulties [45]. In an effort to help advance the growing research literature on resilience among PSP [46], we also administered the Brief Resilience Scale (BRS) to measure clients’ resilience to life stressors [47]. Finally, the Five Facet Mindfulness Questionnaire (FFMQ-15) was administered to measure the degree to which clients engage in 5 aspects of mindfulness (ie, observing, describing, acting with awareness, nonjudging of inner experience, and nonreactivity to inner experience) [48].

#### Patient Health Questionnaire-9

The PHQ-9 is a 9-item self-report measure of the frequency of symptoms of depression in the past 2 weeks. Items are rated on a 4-point Likert-type scale ranging from 0 (not at all) to 3 (nearly every day). Higher scores are indicative of greater symptoms of depression. The PHQ-9 has demonstrated good sensitivity, specificity, and convergent validity [49,50]. Reliability was good to excellent for this study (range of before and after treatment, α=.78-.85; ω=0.79-0.85).

#### Generalized Anxiety Disorder-7

The GAD-7 is a 7-item self-report measure of the frequency of anxiety symptoms in the past 2 weeks. Items are rated on a 4-point Likert-type scale ranging from 0 (not at all) to 3 (nearly every day). Higher scores are indicative of greater symptoms of anxiety. The GAD-7 has demonstrated good internal consistency (α=.89) and good test-retest reliability (*r*=0.83) [42]. The reliability was good to excellent for this study (α=.87-.91; ω=0.87-0.92).

#### Posttraumatic Stress Disorder Checklist for the DSM-5

The PCL-5 is a 20-item measure of each of the 4 clusters of PTSD (ie, intrusive thoughts, avoidance, negative alterations in mood, and alterations in arousal and reactivity). Items are rated on a 5-point Likert-type scale ranging from 0 (not at all) to 4 (extremely). Higher scores are indicative of greater symptoms of PTSD. The PCL-5 has demonstrated strong diagnostic utility within the general population [51] as well as strong test-retest reliability (*r*=0.82) and high internal consistency (ɑ=.94) [43]. Reliability was good to excellent for this study (α=.85-.95; ω=0.94-0.95).

#### Dimensions of Anger Reaction Scale-5

The DAR-5 is a 5-item self-report measure of the dimensions of anger reactions, especially in stressful situations, over the past 4 weeks. Items are rated on a 5-point Likert-type scale ranging from 1 (none or almost none of the time) to 5 (all or almost all of the time). Higher scores are indicative of greater levels of distress. The DARS-5 has demonstrated convergent and discriminant validity [44,52] as well as high internal consistency (α=.95) [44]. Reliability was good to excellent for this study (α=.88; ω=0.89-0.90).

#### Insomnia Severity Index

The ISI is a 7-item self-report measure designed to assess difficulties with sleep and insomnia [45]. Frequency items regarding how often someone experienced a problem in the last 2 weeks are rated on a 5-point Likert-type scale ranging from 0 (none) to 4 (very severe). Items regarding satisfaction with sleep, noticeability of sleep problems, worries about sleep, and interference with daily functioning are rated on a 5-point Likert-type scale, with higher scores indicating greater sleep difficulties. The ISI has demonstrated adequate concurrent validity and good internal consistency (α=.74 [45]).

#### Brief Resilience Scale

The BRS is a 6-item self-report measure of resilience. Items are rated on a 5-point Likert-type scale ranging from 1 (strongly disagree) to 5 (strongly agree). Higher scores are indicative of higher levels of resilience. The BRS has demonstrated good validity and good to excellent internal consistency (α=.80-.91) [47]. Reliability was good to excellent for this study (α=.87-.91; ω=0.87-0.91).

####  Five Facet Mindfulness Questionnaire

The FFMQ-15 is a 15-item short-form self-report mindfulness measure designed to assess 5 facets of mindfulness using 5 subscales: observing, describing, acting with awareness, nonjudging of inner experience, and nonreactivity to inner experience. Statements are rated on a 5-point Likert-type scale ranging from 1 (never or very rarely true) to 5 (very often or always true). Higher scores are indicative of higher levels of mindfulness. The FFMQ-15 has good convergent validity with the FFMQ-39 [48]. Reliability for the total scale was good for this study (α=.79-.85; ω=0.80). Subscale reliability for this study was also good (α=.77-.90; ω=0.81-0.91).

#### Treatment Use and Satisfaction

Each week during the course, clients were asked how much time they spent practicing mindfulness meditation throughout the week in minutes and how many days they practiced. At 8 weeks postenrollment, we administered a bespoke questionnaire assessing satisfaction with the mindfulness meditations and the intervention overall.

### Posttreatment Semistructured Interview

After completing their 8-week measures, clients were invited to participate in semistructured telephone interviews to discuss their perspectives on the mindfulness meditations and the course in general. Invitations were extended upon completion of the course to ensure that clients had sufficient opportunities to review all course materials. Invitations continued until 12 interviews were conducted, which was deemed sufficient given the objectives and the exploratory nature of this study, and this is consistent with prior recommendations [53,54]. Interviews were conducted by a clinician and a research assistant and included questions such as “What parts of the course did you find to be the most helpful? Why?” and “Were there parts of the meditation that you did not like? Why?” Interviews ranged from 10 to 20 minutes and were recorded for later transcription and analysis. The clients were asked to provide both positive and negative constructive feedback on the mindfulness meditations. Informed consent was obtained before the commencement of each interview and confirmed verbally before each recording was initiated. Table 1 shows a summary of all measures used.

**Table 1:** Summary of measures.

Outcome	Instrument	Description	Data collection time points
Sociodemographics	Web-based questionnaire	Self-report web-based questionnaires with questions about gender, province, PSP^a^ sector, ethnicity, and age	Web-based screening
Depression	PHQ-9^b^	9-item self-report measure of symptoms of depression	Web-based screening; 8 weeks postenrollment
Anxiety	GAD-7^c^	7-item self-report measure of symptoms of anxiety	Web-based screening; 8 weeks postenrollment
Posttraumatic stress	PCL-5^d^	20-item self-report measure of symptoms of posttraumatic stress	Web-based screening; 8 weeks postenrollment
Anger	DAR-5^e^	5-item self-report measure of dimensions of anger reactions	Web-based screening; 8 weeks postenrollment
Insomnia	ISI^f^	7-item self-report measure of symptoms of insomnia	Web-based screening; 8 weeks postenrollment
Resilience	BRS^g^	6-item self-report measure of resilience	Web-based screening; 8 weeks postenrollment
Mindfulness	FFMQ-15^h^	15-item self-report measure of 5 facets of mindfulness	Web-based screening; 8 weeks postenrollment
Treatment use and satisfaction	Web-based questionnaire	Self-report web-based questionnaires with questions about treatment use, treatment adherence, and satisfaction with treatment	8 weeks postenrollment
Perspectives on the mindfulness meditations and the course	Posttreatment semistructured interview	Semistructured interview with a trained researcher inquiring about perspectives on mindfulness and the course	10 weeks postenrollment

^a^PSP: public safety personnel.

^b^PHQ-9: Patient Health Questionnaire-9.

^c^GAD-7: Generalized Anxiety Disorder-7.

^d^PCL-5: Posttraumatic Stress Disorder Checklist for the *DSM-5*.

^e^DAR-5: Dimensions of Anger Reaction Scale-5.

^f^ISI: Insomnia Severity Index.

^g^BRS: Brief Resilience Scale.

^h^FFMQ-15: Five Facet Mindfulness Questionnaire.

### Analyses

#### Quantitative Analyses

SPSS (version 26; IBM Corp) was used to conduct the quantitative analyses. Data were deidentified before the analysis. Descriptive statistics were used to describe the sample’s demographic and clinical characteristics, engagement in mindfulness meditation practice, and satisfaction with the mindfulness meditations and the course in general. Completer analysis was used (ie, we did not impute missing data), as is common in pilot studies [55]. Paired sample *t* tests (2-tailed) were conducted to measure changes from baseline to posttreatment in the total scores on the GAD-7, PHQ-9, PCL-5, DAR-5, FFMQ-15, ISI, and BRS. Partial correlations were used to test for relationships between posttreatment measures (as measured by the GAD-7, PHQ-9, DAR-5, FFMQ-15, ISI, and BRS) and minutes spent meditating, when controlling for pretreatment measures.

#### Qualitative Analyses

Client interview data were deidentified and analyzed using NVivo 12 (Lumivero). The data were analyzed using a directed content analysis approach [56]. An initial codebook was developed by CAL to align with the questions posed during the semistructured interview. The data were then grouped into categories by CAL using a realist approach, whereby client data were treated as their descriptions of reality [57]. New codes were created by CAL when the data did not fit into the preexisting codes. The codebook and data were independently reviewed by JDB, a research associate with extensive experience in qualitative research. CAL and JDB convened to address conflicts in the codebook and continued discussions until a consensus was reached.

## Results

### Client Flow and Demographics

**Figure 1:**
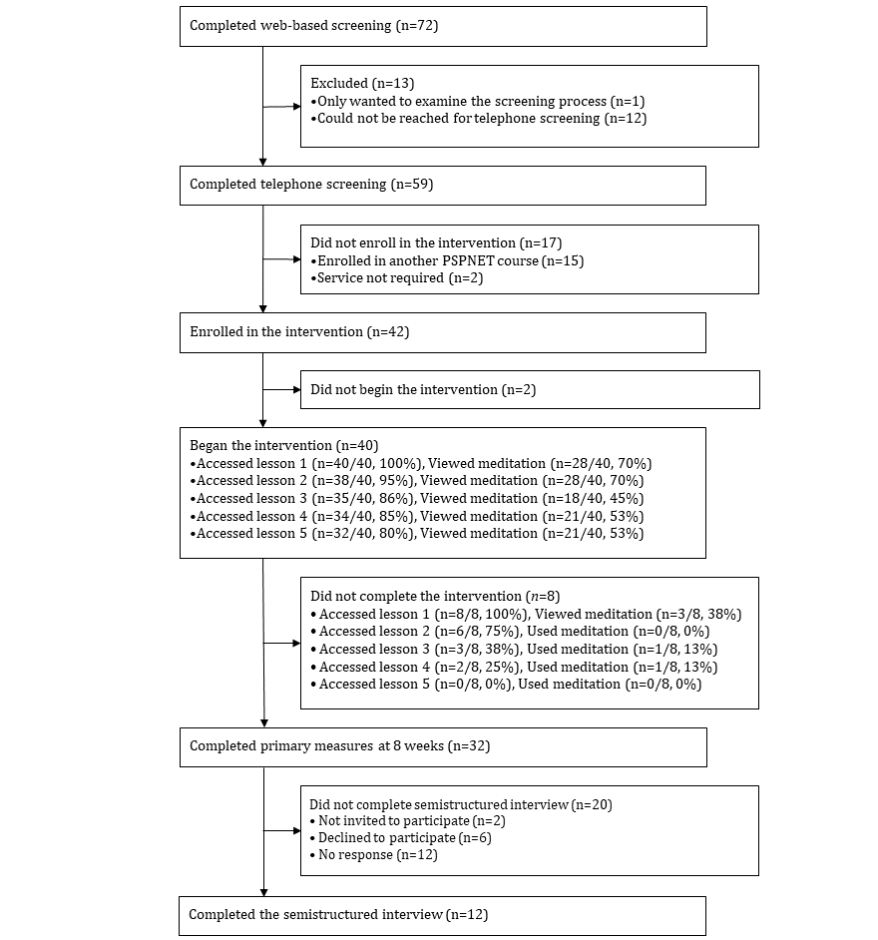
Client flow diagram.

Figure 1 shows the flow of clients through this study, with 40 clients enrolling in and initiating the course and 32 (80%) clients completing the outcome measures. The mean age of the clients was 40.57 (SD 9.75) years. Most clients identified as White (37/40, 93%) and women (26/40, 65%) and reported currently residing in Saskatchewan (27/40, 68%). Most clients (22/40, 55%) reported that they were police, although other occupational groups were also represented (Table 2). In total, 30% (11/40) of the clients had scores above the clinical cut-off on the PHQ-9, 27% (11/40) above the cut-off on the GAD-7, 15% (6/40) above the cut-off on the PCL-5, and 45% (18/40) screened positive for at least 1 mental disorder. In terms of program completion at 8 weeks, 80% (32/40) of the clients had completed all lessons. On average, clients sent 5.42 (SD 3.57) messages to their therapists and received 9.48 (SD 3.18) messages. They also had 2.39 (SD 3.13) phone calls with therapists.

**Table 2:** Clients’ demographic characteristics and occupations (N=40).

Characteristics			Clients, n (%)
**Gender**			
	Women	26 (65)	
	Men	14 (35)	
**Province**			
	Saskatchewan	27 (68)	
	Other province (eg, Prince Edward Island, Alberta, New Brunswick, Nova Scotia, Nunavut, and Ontario)^a^	13 (33)	
**PSP^b^ sector**			
	Police	22 (55)	
	Fire	5 (13)	
	Corrections	4 (10)	
	Communications (eg, 911 and dispatch)	4 (10)	
	Other	5 (13)	
**Ethnicity**			
	Ethnic minority (eg, First Nations, Inuit, and Metis; Asian; Middle Eastern; Black; and South Asian)^a^	3 (8)	
	White	37 (93)	
**Age (y)**			
	20-29	6 (15)	
	30-39	10 (25)	
	40-49	16 (40)	
	≥50	7 (17)	

^a^Cells are merged owing to the small cell size to protect confidentiality.

^b^PSP: public safety personnel.

### Meditation Use

No prospective clients reported participating in regular mindfulness practice at the time of their enrollment. Figure 1 shows that of the 40 clients, 28 (70%) viewed the grounding meditation in lesson 1, 28 (70%) viewed the loving kindness meditation in lesson 2, 18 (45%) viewed the meditation on awareness of breath in lesson 3, 21 (53%) viewed the meditation on awareness of the 5 senses in lesson 4, and 21 (53%) viewed the body scan meditation in lesson 5, as tracked through automatic computer system logs. Meditations were available for download; as such, some clients may have listened to the meditations more often than indicated by the system. Clients who completed the course tended to spend more time on the meditations overall, although this difference was not statistically significant (*P*>.16). Table 3 shows the mean number of text views and audio listens for each meditation and suggests that clients demonstrated a preference for reviewing the text over listening to the audio. Table 3 shows that the clients who completed the course accessed the written meditations an average of 4.91 (SD 2.91) times. Table 4 shows the self-reported number of times meditating and the number of minutes spent meditating and shows that those who meditated, meditated on average 5.67 times for a total of 28.48 minutes of meditation. As shown in Table 4, there was wide variability in days meditating (0-28 days) and minutes meditating (0-290 minutes) over the course. Of note, 9 clients opened meditation materials but reported that they did not use those materials.

**Table 3:** Web-based meditation views and listens.

	All clients (N=40), mean (SD; range)		Course completers (n=32), mean (SD; range)		Course noncompleters (n=8), mean (SD; range)	
	Text views	Audio listens	Text views	Audio listens	Text views	Audio listens
Lesson 1	1.25 (1.19; 0-4)	0.73 (0.93; 0-3)	1.39 (1.22; 0-4)	0.76 (0.97; 0-3)	0.57 (0.79; 0-2)	0.57 (0.79; 0-2)
Lesson 2	1.05 (0.96; 0-4)	0.48 (0.85; 0-4)	1.15 (0.97; 0-4)	0.58 (0.90; 0-4)	0.57 (0.79; 0-2)	0.00 (0; 0-0)
Lesson 3	0.60 (0.84; 0-4)	0.43 (0.75; 0-3)	0.67 (0.85; 0-4)	0.48 (0.80; 0-3)	0.29 (0.76; 0-2)	0.00 (0; 0-1)
Lesson 4	0.73 (0.82; 0-3)	0.18 (0.38; 0-1)	0.85 (0.83; 0-3)	0.21 (0.42; 0-1)	0.15 (0.38; 0-1)	0.15 (0.38; 0-0)
Lesson 5	0.78 (0.92; 0-3)	0.25 (0.44; 0-1)	0.85 (0.87; 0-3)	0.30 (0.47; 0-1)	0.43 (1.13; 0-3)	0.00 (0; 0-0)
Total	4.40 (3.14; 0-12)	2.05 (2.25; 0-9)	4.91 (2.91; 0-12)	2.33 (2.34; 0-9)	2.00 (3.27; 0-9)	0.71 (1.11; 0-3)

**Table 4:** Days and minutes spent meditating.

	All clients (N=40), mean (SD; range)			Course completers (n=32), mean (SD; range)			Course noncompleters (n=8), mean (SD; range)	
	Days meditated	Minutes meditated	Days meditated		Minutes meditated	Days meditated		Minutes meditated
Week 2	0.57 (1.26; 0-6)	1.68 (6.90; 0-40)	0.70 (1.36; 0-6)		2.03 (7.57; 0-40)	0 (0; 0-0)		0 (0; 0-0)
Week 3	0.45 (0.78; 0-3)	3.25 (6.94; 0-20)	0.55 (0.83; 0-3)		3.94 (7.48; 0-20)	0 (0; 0-0)		0 (0; 0-0)
Week 4	0.88 (1.42; 0-6)	3.13 (10.66; 0-60)	1.06 (1.50; 0-6)		3.79 (11.66; 0-60)	0 (0; 0-0)		0 (0; 0-0)
Week 5	0.48 (1.01; 0-3)	4.20 (10.75; 0-60)	0.58 (1.09; 0-3)		5.09 (11.67; 0-60)	0 (0; 0-0)		0 (0; 0-0)
Week 6	0.80 (1.57; 0-6)	4.38 (12.26; 0-60)	0.97 (1.69; 0-6)		5.30 (13.34; 0-60)	0 (0; 0-0)		0 (0; 0-0)
Week 7	0.75 (1.50; 0-6)	4.00 (14.99; 0-70)	0.91 (1.65; 0-6)		4.85 (16.42; 0-70)	0 (0; 0-0)		0 (0; 0-0)
Week 8	0.75 (1.60; 0-6)	2.88 (8.00; 0-40)	0.91 (1.72; 0-6)		3.48 (8.70; 0-40)	0 (0; 0-0)		0 (0; 0-0)
Total	4.68 (6.50; 0-28)	23.50 (49.07; 0-290)	5.67 (6.76; 0-28)		28.48 (52.80; 0-290)	0 (0; 0-0)		0 (0; 0-0)

### Changes in Symptoms, Resilience, and Mindfulness

The 32 clients who completed the 8-week measures reported statistically significant reductions in total scores on the GAD-7, PHQ-9, PCL-5, and DAR-5 (all *P*<.001) and a statistically significant increase in BRS total scores (*P*=.01). No statistically significant change in ISI total scores was observed. Clients who completed the 8-week measures reported statistically significant increases in overall FFMQ-15 scores, with examination of the subscales revealing changes in mindfulness nonjudging and nonreactivity scores but not describing, acting with awareness, or observing. Time spent meditating was not associated with posttreatment measures while controlling for pretreatment measures (all *P*=.09-.67). The details are presented in Table 5.

**Table 5:** Changes in outcome measures.

Measures	Pretreatment score, mean (SD)	Posttreatment score, mean (SD)	*t* test (*df*)	*P* value	Cohen *d*
GAD-7^a^	7.88 (6.13)	1.48 (4.07)	−3.74 (32)	<.001	−1.23
PHQ-9^b^	8.81 (5.41)	5.27 (3.51)	−5.10 (32)	<.001	−0.78
PCL-5^c^	20.12 (15.61)	10.42 (10.48)	−6.13 (32)	<.001	−0.74
DAR-5^d^	9.85 (4.23)	7.40 (2.84)	−3.87 (32)	<.001	−0.67
ISI^e^	10.42 (6.06)	9.09 (4.59)	−1.70 (32)	.02	−0.30
BRS^f^	3.15 (0.74)	3.42 (0.71)	2.72 (32)	.01	−0.46
FFMQ-15^g^	38.27 (8.25)	40.58 (7.40)	3.51 (32)	<.001	0.47
Acting with awareness	9.52 (2.53)	9.48 (2.51)	−0.11 (32)	.91	0.03
Describing	9.34 (3.14)	9.90 (2.85)	1.57 (32)	.13	0.30
Nonjudgmental	10.00 (2.93)	10.85 (1.43)	2.68 (32)	.01	0.42
Nonreactivity	9.36 (2.83)	10.33 (2.16)	2.55 (32)	.02	0.44
Observing	8.48 (2.98)	8.79 (2.87)	2.72 (32)	.40	0.15

^a^GAD-7: Generalized Anxiety Disorder-7.

^b^PHQ-9: Patient Health Questionnaire-9.

^c^PCL-5: Posttraumatic Stress Disorder Checklist for the *DSM-5*.

^d^DAR-5: Dimensions of Anger Reaction Scale-5.

^e^ISI: Insomnia Severity Index.

^f^BRS: Brief Resilience Scale.

^g^FFMQ-15: Five Facet Mindfulness Questionnaire.

### Treatment Satisfaction

The 32 clients who completed the 8-week measures generally reported feeling satisfied with the course. The 27 clients who accessed the mindfulness meditations also generally reported that they were satisfied with the mindfulness meditations. Most clients reported that they would recommend the intervention to a friend (32/32, 100%) and that they would recommend the mindfulness meditations to a friend (25/32, 93%). Table 6 provides details.

**Table 6:** Treatment satisfaction (n=32).

			Intervention (n=32), n (%)		Mindfulness meditations (n=27), n (%)
**Recommend to a friend**					
	Yes	32 (100)		25 (93)	
	No	0 (0)		2 (7)	
**Satisfaction**					
	Very dissatisfied	0 (0)		0 (0)	
	Dissatisfied	1 (3)		1 (3)	
	Neutral	4 (13)		11 (41)	
	Satisfied	15 (47)		10 (37)	
	Very satisfied	12 (38)		5 (19)	
**Worth the time**					
	Yes	32 (100)		25 (89)	
	No	0 (0)		2 (7)	
**Increased confidence in the ability to manage symptoms**					
	Reduced	0 (0)		0 (0)	
	No change	0 (0)		0 (0)	
	Increased	22 (69)		17 (63)	
	Greatly increased	10 (31)		10 (37)	

### Qualitative Results

Overall, clients reported that the mindfulness meditations were beneficial (Table 7). Key themes from the qualitative analysis included clients liking the simplicity of following the mindfulness meditations, incorporating other strategies into the mindfulness meditations, and the variety of mindfulness meditations that were presented. Suggestions included providing videos alongside the mindfulness meditations for individuals who consider themselves to be more “visual,” providing distinct end points in the mindfulness meditations (eg, a bell chime to indicate when the meditation end), and providing shorter mindfulness meditations to start. Suggestions for technical or presentation changes, including creating an atmosphere for mindfulness meditations to be completed in a group setting, were also provided.

**Table 7:** Results of the qualitative analyses (n=12).

Theme			Endorsing theme, n (%)		Client quotations reflecting the themes
**Tried meditation before beginning the course (n=9, 75%)**					
	Yes, beneficial	7 (78)		“I’d used mindfulness quite a bit in the past [and I] still use it to some extent” [Client 26]	
	Yes, skeptical	2 (22)		“I kinda thought it would be maybe hokey-pokey...um, a little wishy-washy...yeah, I was a little skeptical shall we say” [Client 20]	
Have not tried meditation before beginning the course			3 (25)		N/A^a^
Beneficial for PSP^b^			10 (83)		“It’s nice to, it’s probably a really good idea for most of us, ‘cause I think anybody who is in, ah, policing and-and stuff is, ah, likely type A...go-go-go type of personality...so it’s good to take a step back, focus on what you can control, because everything we deal with is out of our control” [Client 21]
Mindfulness reduced stress and improved relaxation			7 (58)		“I think definitely with stress I found if I was stressing about something in my job or my life or whatever, once I did the meditation usually I would—during meditation I’d be able to reflect on what I was stressed about.” [Client 18]
Mindfulness helped them to slow down and regulate their bodies and emotions			5 (42)		“It was kinda nice ‘cause it just got me to slow down and actually focus on my breathing. And just doing that kinda helped regulate everything else that was going on with my body.” [Client 16]
Mindfulness can be completed on their own time			2 (17)		“I found that helpful to be able to go back and replay them basically whenever I needed.” [Client 36]
Mindfulness reminded them to be gentle with themselves			2 (17)		“It made me talk to myself a little bit differently and be a little bit kinder to myself instead of like, focusing on everything I was doing wrong.” [Client 26]
**Challenges with mindfulness**					
	Feeling uncomfortable sitting with their feelings	8 (67)		“I think most people struggle with quieting their minds and actually taking the time to do it. It seems really painful to sit there and keep refocusing your mind as it wanders.” [Client 20]	
	Difficulty finding motivation, time, and quiet space	8 (67)		“It’s kind of hard sometimes to find quiet time or quiet space in my house.” [Client 18]	
	Technical issues	2 (17)		“I struggled with the meditations because the audio never worked for me.” [Client 32]	
Suggestions for improvement			5 (42)		“For me anything more than five to ten minutes, then I’m off the rails and I can’t concentrate anymore, so shorter is better for me.” [Client 21]

^a^N/A: not applicable.

^b^PSP: public safety personnel.

## Discussion

### Principal Findings

In this study, we explored the feasibility of adding mindfulness meditations to the PSP Wellbeing Course, as there is a gap in the literature with regard to the use of mindfulness meditations among PSP participating in iCBT. Overall, the study suggested that there was highly variable use of mindfulness meditations among PSP in the PSP Wellbeing Course*,* with 70% (28/40) of the clients reviewing the first 2 meditations and then about half of clients (ie, 18/40, 45% to 21/40, 53%) viewing the last 3 meditations. Clients more often looked at the text of the meditations rather than the audio of the meditations. There was significant variability among clients in the use of the meditations, with some clients never using the meditations and others using the meditations 3 to 4 times a week. Similarly, in terms of practice, there was high variability observed, with clients on average practicing 23 minutes a week. The use data alone suggest that the incorporation of mindfulness within iCBT will not be universally used when offered. It is not fully known why some clients did not use the meditations. It is possible that the high amount of content in the PSP Wellbeing Course itself may be a factor in the lower use of mindfulness meditations by some clients (ie, some clients may not have had enough time or willingness to practice mindfulness meditation in addition to other skills taught in the course).

In terms of improvements on measures over 8 weeks, clients in this course reported statistically significant improvements in symptoms of anger, anxiety, depression, PTSD, and insomnia. These findings are consistent with previous results regarding the PSP Wellbeing Course [9]. Although this was not a randomized controlled trial comparing iCBT alone with iCBT enhanced with mindfulness meditations, the results suggest that the addition of mindfulness did not have a marked positive or negative impact on the effectiveness of the course.

Other benefits observed in this study included improved resilience and improved mindfulness scores from pre- to posttreatment. In terms of mindfulness, the results suggested that the course was specifically associated with improvements in nonjudging and nonreactivity mindfulness scores. It appears that the course reduced the frequency with which clients labeled their thoughts and feelings as “good” or “bad” and enhanced client’s ability to detach from thoughts and feelings rather than getting caught up in their thoughts and feelings. The course was not associated with changes in observing (ie, noticing internal and external experiences), describing (ie, being able to express one’s experiences in words), or acting with awareness (ie, attending to present-moment experiences) mindfulness scores. The skills of observing, describing, and acting with awareness may be more specifically associated with the practice of mindfulness, and clients may not have participated in sufficient mindfulness meditations to experience these benefits.

As this was not a randomized controlled trial, we could not determine whether the changes in mindfulness were related to the mindfulness meditations or would have resulted from the PSP Wellbeing Course alone. In general, clients who participated in the interviews on mindfulness meditations reported a number of perceived benefits of mindfulness despite variable engagement with the mindfulness meditations. Most clients reported that mindfulness meditation helped reduce stress and improved relaxation and that mindfulness meditation can be beneficial with practice. Mindfulness has previously been associated with stress reduction and improved relaxation, among other benefits [16,58,59]. Clients also reported that they liked that other skills (eg, controlled breathing and emotion labeling) could be incorporated with the mindfulness meditations.

The interviews shed light on the challenges of offering mindfulness meditations to PSP as part of iCBT. A common challenge with the meditations that clients reported was the difficulty in sitting with their emotions. Clients also commonly reported difficulties with finding time or quiet space in which to complete the mindfulness meditations and reported that the mindfulness meditations were too lengthy. Beginning with shortened meditations that slowly increase in length may be better to help PSP to learn to cultivate mindfulness gradually. Future iterations of the intervention may benefit from including information on how mindfulness meditation can be practiced throughout the day in smaller timeframes to increase the accessibility of mindfulness meditation. A suggestion for improvement included providing shorter mindfulness meditations (eg, 2-3 min) to help people learning mindfulness meditation to work their way up to longer mindfulness meditations and gain increased comfort while sitting with their feelings.

Overall, the high degree of treatment satisfaction across the previous version of the PSP Wellbeing Course and this version enhanced with mindfulness meditation suggests that mindfulness meditations, at the very least, did not markedly decrease course satisfaction or negatively impact clients’ perceptions of the course. Of note, there was also no evidence to suggest that this version of the course impacted course completion. At 8 weeks, 80% (32/40) of the clients in this trial had completed all 5 lessons. Similarly, at 8 weeks, 77% of the clients in a prior study of the original PSP Wellbeing Course had completed the course [9]. In terms of therapist engagement, clients in this course sent an average of 5.24 emails and received 9.48 emails from their therapists. In the previous version of the course, on average, clients sent 4.98 (SD 5.53) messages to their therapists and received 9.80 (SD 4.71) messages [9]. Descriptively, treatment satisfaction was lower for mindfulness meditations than for other aspects of the course (ie, lesson material, meditations, and additional resources). The lower satisfaction with the mindfulness meditations may indicate that work is needed to improve the meditations and that some PSP are not open to practicing mindfulness.

Our sample of clients, who voluntarily enrolled in a therapist-guided iCBT course, may have been more interested in CBT skills than mindfulness skills. The large variation in the quality of publicly available mindfulness meditation programs means the clients in this study may have been justifiably skeptical of mindfulness meditation or unsure about the potential benefits of mindfulness meditation [60].

Of note, in this study, no statistically significant relationships were observed between time spent practicing mindfulness meditations and reductions in symptoms, which contrasts with previous evidence that increased time spent meditating is associated with increased symptom reduction [23]. This discrepancy may be a result of the small sample size and having inadequate power to detect effects. This discrepancy may also be explained by the finding that the intervention was already associated with significant reductions in symptoms, and the addition of mindfulness meditations could not meaningfully add to the results. Furthermore, previous studies on PSP and mindfulness appear to focus on the intervention as a whole (ie, the impact of the intervention and mindfulness combined) and have not considered the independent impact of mindfulness on outcomes. It should be noted that the extant research suggests that mindfulness can have positive effects when practiced for 10 minutes daily, but shorter periods can also be associated with benefits [39-41]. Evidence also suggests that the quality of mindfulness meditation may be more important than the amount of mindfulness meditation practiced [61]. Few studies have reported the actual time their clients reported practicing mindfulness [61]. In this study, it may have been preferable to assess the quality of practice rather than the minutes practiced.

### Limitations

This study has important limitations that will help to inform future research. First, the sample size of the study was small; however, the detailed data collected allowed for an understanding of the usability and credibility of mindfulness meditation in a sample of PSP seeking treatment. The detailed data also allow for iterative improvements to the intervention*,* such as providing increased psychoeducation on mindfulness meditation and introducing ultrabrief meditations for clients who want to work for longer periods*.* Future studies on iCBT should be conducted using larger samples of PSP from diverse communities across Canada (and internationally), as this study is not generalizable cross-culturally. It should also be noted that the clients in this study overall had variable clinical symptoms. On average, the scores of this particular subsample suggested that clients had milder symptoms of depression, anxiety, anger, and PTSD, although 30% (11/40) of the clients had scores above the clinical cut-off on the PHQ-9, 27% (11/40) above the cut-off on the GAD-7, 15% (6/40) above the cut-off on the PCL-5, and 45% (18/40) screened positive for at least 1 of the. aforementioned mental disorders. It is possible that the lower symptom severity overall in the sample may have impacted the relationship between minutes meditating and outcomes owing to floor effects. It is also notable that in this sample, there were more women than men, which may have affected the findings. Second, this study does not contain a randomized design and involves short-term follow-up; thus, it is descriptively compared with the previously published trial on the PSP Wellbeing Course. Future studies of the program could benefit from a randomized design and a larger sample size to evaluate potential differences in experiences between versions of the course. In addition, such studies could explore how the course compares with control conditions. Third, only a subsample of the clients was interviewed, and the experiences of those who did and did not consent to an interview could have differed (eg, clients with more favorable attitudes toward the mindfulness meditations may have been more likely to consent to an interview). Fourth, our ability to determine whether time spent meditating predicted outcome measure changes may have been limited by the low engagement from clients in the mindfulness meditations. Future studies should consider means to encourage mindfulness meditation use without clients feeling forced to complete the exercises.

### Clinical Implications and Future Directions

This study has several important potential implications. First, results from this study replicated previous results regarding the PSP Wellbeing Course, indicating that the intervention is associated with statistically significant reductions in symptoms of anxiety, depression, PTSD, and anger with medium to large effect sizes [62]. Contributing to the literature on iCBT for PSP, the study showed that improvements were also observed in resilience and mindfulness with this version of the course.

Use data showed that a significant percentage of clients used the mindfulness meditations. The information gathered from the interviews indicated that clients enjoyed that the mindfulness meditations could be incorporated with other strategies. Therapists may be able to help support future clients in understanding what works for them and how to incorporate their existing strategies with mindfulness meditation to achieve increased benefits. Future programs may benefit from increasing psychoeducation regarding the potential discomfort associated with mindfulness meditation and encourage clients to consider practicing more to allow them to get used to experiencing emotions. Clients also made suggestions for improvement. The mindfulness meditations may be offered after the completion of the course as an additional resource for clients who may be interested in pursuing the meditations. Additional research is needed to identify who can benefit from mindfulness meditations and to ensure that those who do not benefit are not distracted from the rest of the course content in attempting to complete mindfulness activities. One potential solution is to include mindfulness meditations as an optional component, allowing interested individuals to use mindfulness within the course while not distracting individuals who are not interested in mindfulness.

### Conclusions

To the best of our knowledge, this is the first study to assess web-based mindfulness meditations as an adjunct to a preexisting iCBT intervention among PSP. The incorporation of mindfulness meditation was largely acceptable to many clients enrolled in the intervention, although our findings were limited by variable client engagement with the mindfulness meditations. The mindfulness meditations were used by approximately half of the clients, and the clients who used them reported that they enjoyed the meditations. The addition of mindfulness meditations did not appear to remarkably affect the overall engagement, satisfaction, or outcomes of the course. Clients also expressed several challenges and suggestions for improvement, which represent opportunities to improve mindfulness meditations and iCBT for PSP in general. The presentation of shortened mindfulness meditations may serve as an important adjustment to allow PSP to experience the benefits of mindfulness while maintaining their busy schedules. The results of this study suggest that mindfulness meditations, when offered along with iCBT, may be an acceptable intervention for Canadian PSP. Future research is required to further explore how best to incorporate mindfulness into iCBT for PSP and the potential benefits of doing so now that we have demonstrated partial feasibility.

## Abbreviations

BRS: Brief Resilience Scale
CBT: cognitive behavioral therapy
DAR-5: Dimensions of Anger Reaction Scale-5
FFMQ-15: Five Facet Mindfulness Questionnaire
GAD-7: Generalized Anxiety Disorder-7
iCBT: internet-delivered cognitive behavioral therapy
ISI: Insomnia Severity Index
PCL-5: Posttraumatic Stress Disorder Checklist for the *DSM-5*
PHQ-9: Patient Health Questionnaire-9
PSP: public safety personnel
PTSD: posttraumatic stress disorder

## Data Availability

As part of the consent form, participants were informed that their data would only be shared with members of the research team. Therefore, we are unable to share data.
